# Expression of ODC Antizyme Inhibitor 2 (AZIN2) in Human Secretory Cells and Tissues

**DOI:** 10.1371/journal.pone.0151175

**Published:** 2016-03-10

**Authors:** Tiina Rasila, Alexandra Lehtonen, Kristiina Kanerva, Laura T. Mäkitie, Caj Haglund, Leif C. Andersson

**Affiliations:** 1 Department of Pathology, University of Helsinki, Helsinki, Finland; 2 Institute of Biomedicine, Anatomy, University of Helsinki, Finland; 3 Research Programs Unit, Translational Cancer Biology, University of Helsinki, Helsinki, Finland; Universidad de Malaga, SPAIN

## Abstract

Ornithine decarboxylase (ODC) antizyme inhibitor 2 (AZIN2), originally called ODCp, is a regulator of polyamine synthesis that we originally identified and cloned. High expression of ODCp mRNA was found in brain and testis. We reported that AZIN2 is involved in regulation of cellular vesicle transport and / or secretion, but the ultimate physiological role(s) of AZIN2 is still poorly understood. In this study we used a peptide antibody (K3) to human AZIN2 and by immunohistochemistry mapped its expression in various normal tissues. We found high expression in the nervous system, in type 2 pneumocytes in the lung, in megakaryocytes, in gastric parietal cells co-localized with H,K-ATPase beta subunit, in selected enteroendocrine cells, in acinar cells of sweat glands, in podocytes, in *macula densa* cells and epithelium of collecting ducts in the kidney. The high expression of AZIN2 in various cells with secretory or vesicle transport activity indicates that the polyamine metabolism regulated by AZIN2 is more significantly involved in these events than previously appreciated.

## Introduction

The polyamines, putrescine, spermidine and spermine are organic polycations known to be involved in regulation of many fundamental cellular functions like proliferation, differentiation, malignant transformation and apoptosis [1,2]. The ultimate molecular mechanism(s) by which polyamines exert their activity is still however incompletely understood [3]. Ornithine decarboxylase (ODC), which decarboxylates ornithine to generate putrescine, is the rate-limiting enzyme of polyamine synthesis. Elevated ODC activity is typically found in rapidly proliferating cells and in neoplastic tissue. ODC is a transcriptional target of the c-myc oncogene [4] but has also itself oncogenic properties. We originally reported that overexpression of human ODC cDNA in NIH3T3 cells induced their malignant transformation [5] with ability to form tumors in athymic mice [6]. Given the cellular impact of ODC its activity is under strict transcriptional and posttranslational regulation [7]. A sizeable portion of cellular ODC is bound as catalytically inactive monomers to proteins called antizymes (AZ) [8,9]. Four members of human antizymes have been identified out of which AZ1 is ubiquitously expressed. AZ1 directs ODC for proteosomal degradation independently of ubiquitination and also inhibits cellular uptake of polyamines.

Antizyme inhibitors (AZIN) are AZ antagonists. They are homologous to ODC but devoid of catalytic activity. Monomeric AZINs bind AZs with higher affinity than ODC thereby releasing sequestered ODC to form catalytically active dimers [8,10]. In addition to releasing ODC the binding of AZ by AZIN may also reduce the degradation of ODC.

AZIN, now called AZIN1, was first identified in 1982 by Fujita et al. [11] Accumulated data indicate that AZIN1 is functionally linked to normal and malignant cell proliferation. Forced overexpression of AZIN1 in NIH3T3 cells induces malignant transformation like that seen with overexpression of ODC [12]. Gene amplifications of AZIN1 have been found in various human neoplasms including cancer in the breast, ovary and prostate [13].

We originally identified and cloned the second form of AZIN initially called ODC paralog (ODCp). ODCp was found to potentially occur in at least eight alternative spliced forms. The highest levels of ODCp mRNA was found in the brain and testis. Since we noticed that cysteine 360, which is critical for ODCs catalytic activity, was substituted by valine in ODCp we suggested in the original report that ODCp represents a novel form of ODC antizyme inhibitor [14]. The antizyme inhibitory activity of ODCp was subsequently demonstrated in mouse [15] and human [15,16] and is now called AZIN2.

Physiologically, AZIN2 appears most abundantly in differentiated resting cells or in cells with slow turnover. By immunohistochemistry we found robust expression of AZIN2 in the brain along the neural axons and dendrites in a granular or vesicular pattern [17]. An intriguing finding was the elevated expression of AZIN2 in the brain of Alzheimer patients. This may be of relevance for higher content of polyamines present in the brain in Alzheimer’s disease [18].

The antibody used for staining of AZIN2 in the brain reacted mainly with Leydig cells in normal testis with only weak reactivity in the germinal epithelium [19].

By the use of a temperature-sensitive mutant of viral glycoprotein, VSVG3^ts045^, we showed that intact AZIN2 regulates intracellular vesicle transport in MCF/7 breast cancer cells [20].

We also reported expression of AZIN2 in human normal mast cell and mastocytomas. Downregulation of AZIN2 expression in mast cell selectively blocked stimulated release of serotonin without appreciable effect on histamine release [21].

Lopez-Garcia et al. created AZIN2 hypomorphic mice and found evidence for regulatory influence of AZIN2 on secretion of insulin from pancreatic islets [22].

To obtain a comprehensive view of the distribution of AZIN2 in human cells and tissues we used AZIN2 peptide antibodies made in rabbits and mapped its endogenous expression by immunohistochemistry.

## Materials and Methods

Paraffin blocks containing normal human tissues were collected from the archives of the Department of Pathology of University of Helsinki and HUSLAB according to the local legislation. The study was approved by the Surgical Ethics Committee of Helsinki University Hospital (Dnro HUS 226/E6/06, extension TMK02 §66 17.4.2013), and the National Supervisory Authority of Welfare and Health (Valvira Dnro 10041/06.01.03.01/2012). Freshly cut 4-μm tissue sections were deparaffinized in xylene and rehydrated through a gradually decreasing concentration of ethanol to distilled water. Slides were treated in a PreTreatment module (Lab Vision Corp., Fremont, CA, USA) in Tris-EDTA (pH 9) buffer for 20 min at 98°C for antigen retrieval. Immunohistochemical staining of sections was performed in an Autostainer 480 (Lab Vision Corp., Fremont, CA, USA) by the Dako REAL EnVision Detection system, Peroxidase/DAB+, Rabbit/Mouse (Dako, Glostrup, Denmark). Sections were incubated with anti-AZIN2 antibody (K3) for one hour at room temperature. *In situ* hybridization with sense and anti-sense AZIN2 cRNA probes was performed using a Ventana Discovery Stainer Platform for automated hybridization. The design and making of the diogenine-UTP labeled cRNA probes and visualization of the hybridization have been described in detail [17,19].

We raised rabbit antibodies to different peptides representing unique portions of the AZIN2 molecule [17,19,21]. In this study we have used antibody K3 that was made against a peptide (STRDLLKELTLGASQATT) representing the amino acids 18–35 of AZIN2. Branched peptides were synthesized with an automated multiple peptide synthesizer (MultiPep; Intavis Bioanalytical Instruments AG, Cologne, Germany) using Fmoc-chemistry and purified with HPLC using a Supelco Discovery Biowide Pore C18 column (Supelco, Bellefonte, PA, USA). The main peak was collected, lyophilized and used as antigens for immunizations. The antibody was produced at the Viikki Laboratory Animal Centre, University of Helsinki, Finland. All animals were handled in strict accordance with good animal practice as defined by the relevant Finnish animal welfare bodies, and the European Communities Council directive (86/609/EEC). All animal work was approved by the Animal Experiment Board of the State Provincial Office of Southern Finland (Reference number HY176-02) in accordance with national legislation. Validation of the specificity of the antibody called K3 has been reported [17,19,21]. The antibody showed no cross-reactivity with ODC or with AZIN1 [17,19,21]. The K3 antibody was used at a dilution of 1:500–1:800 for immunohistochemistry and 1:150 for indirect immunofluorescence. Pre-immune serum from the same rabbit was used as negative control. The antibodies used for immunohistochemistry and indirect immunofluorescence are listed in Table 1.

**Table 1:** Antibodies for immunohistochemistry (IHC) and immunofluorescence (IF).

Antibody	Clone	Company	Pre-treatment	Dilution
A. Primary antibodies.				
K3	Rabbit	in-house	Tris-HCL (pH 8.5)	1:500–1:800 (IHC) 1:150 (IF)
H,K-ATPase	mAb 2B6	MBL International	Citrate (pH 6.0)	1:100 (IHC) 1:30 (IF)
Serotonin	mAb 5HT-H209	DAKO	Citrate (pH 6.0)	1:20 (IF)
WT1	mAb WT1-562	Novocastra	Citrate (pH 6.0)	1:20 (IF)
Chromogranin A	mAb 319	Upstate Millipore	Citrate (pH 6.0)	1:30 (IF)
Rab27b	Rabbit 13412-1-AP	Proteintech	Citrate (pH 6.0)	1:400 (IHC)
B. Secondary antibodies for indirect immunofluorescence.				
Donkey-anti-rabbit, Alexa Fluor^®^ 555 conjugate	A31572	Invitrogen		1:1000
Donkey-anti-mouse, Alexa Fluor^®^ 488 conjugate	A21202	Invitrogen		1:1000

Photographs of IHC stained sections were taken with an Olympus BX51 microscope equipped with a Nikon DS-U1 camera. IF stainings were photographed with a Zeiss Axiophot2 microscope or with a Leica SP2 confocal microscope.

## Results

### The Nervous System

**Fig 1:**
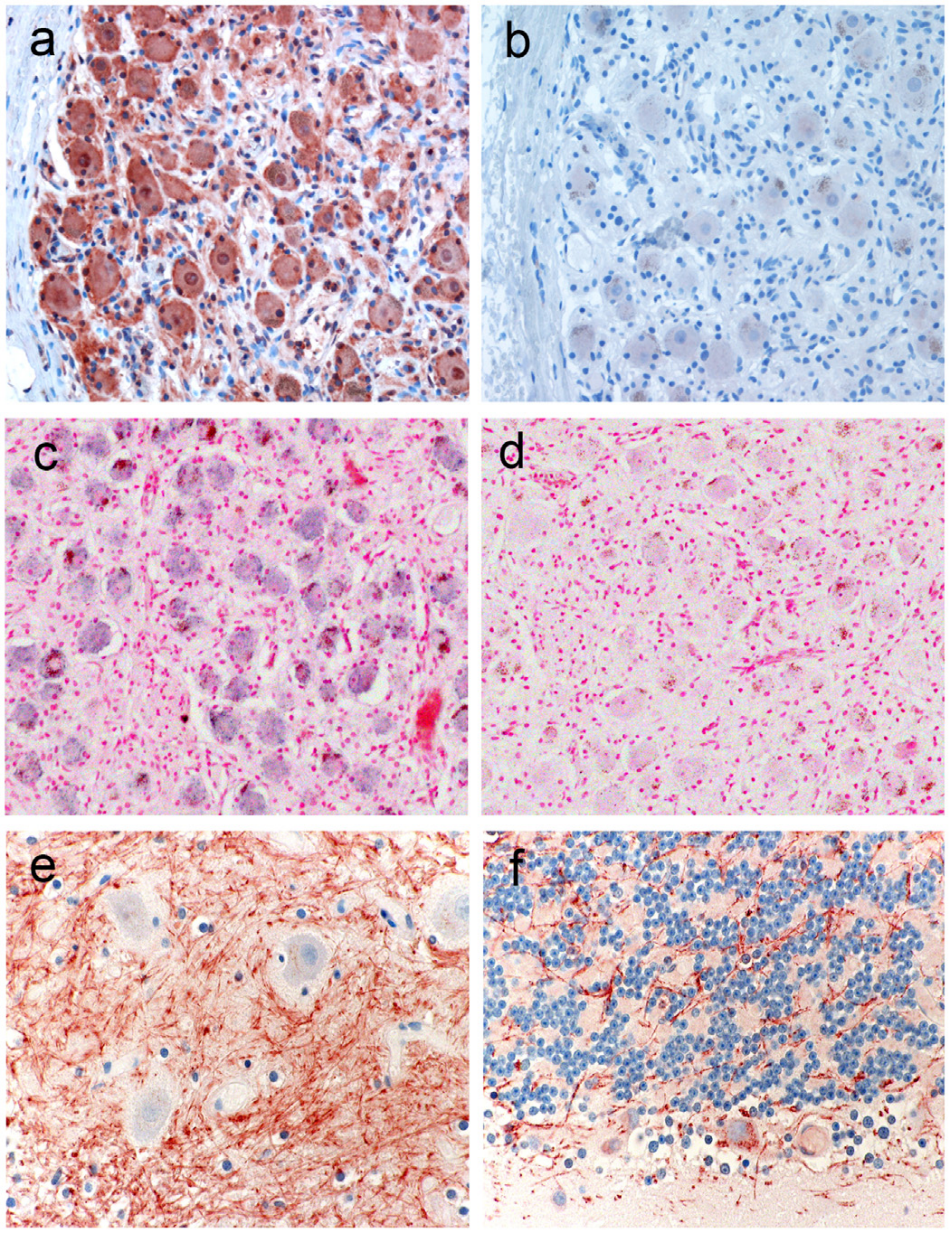
Expression of AZIN2 in neural tissue. Nerve cells in a spinal ganglion stained with K3 (a) and control staining with pre-immunization serum from the same rabbit (b). *In situ* hybridization with sense RNA probe (c) and anti-sense (control) probe (d). Staining of a portion of *Nucleus dentatus* (e) and of the granular cell layer in cerebellum (f).

We have previously reported robust expression of AZIN2 in the brain distributed along neural axons and dendrites in a granular or vesicular pattern [17]. While antibody K3 stained dendrites and axons in the CNS somas of nerve cell bodies in spinal ganglions showed strong reactivity (Fig 1a). The presence of AZIN2 mRNA was confirmed by *in situ* hybridization (Fig 1c). Also the myenteric (Auerbach′s) nerve plexuses in the intestinal wall stained with antibody K3 (not shown). Staining patterns with K3 antibody in the brain stem (Fig 1e) and cerebellum (Fig 1f) are shown.

### Hematopoietic Cells

**Fig 2:**
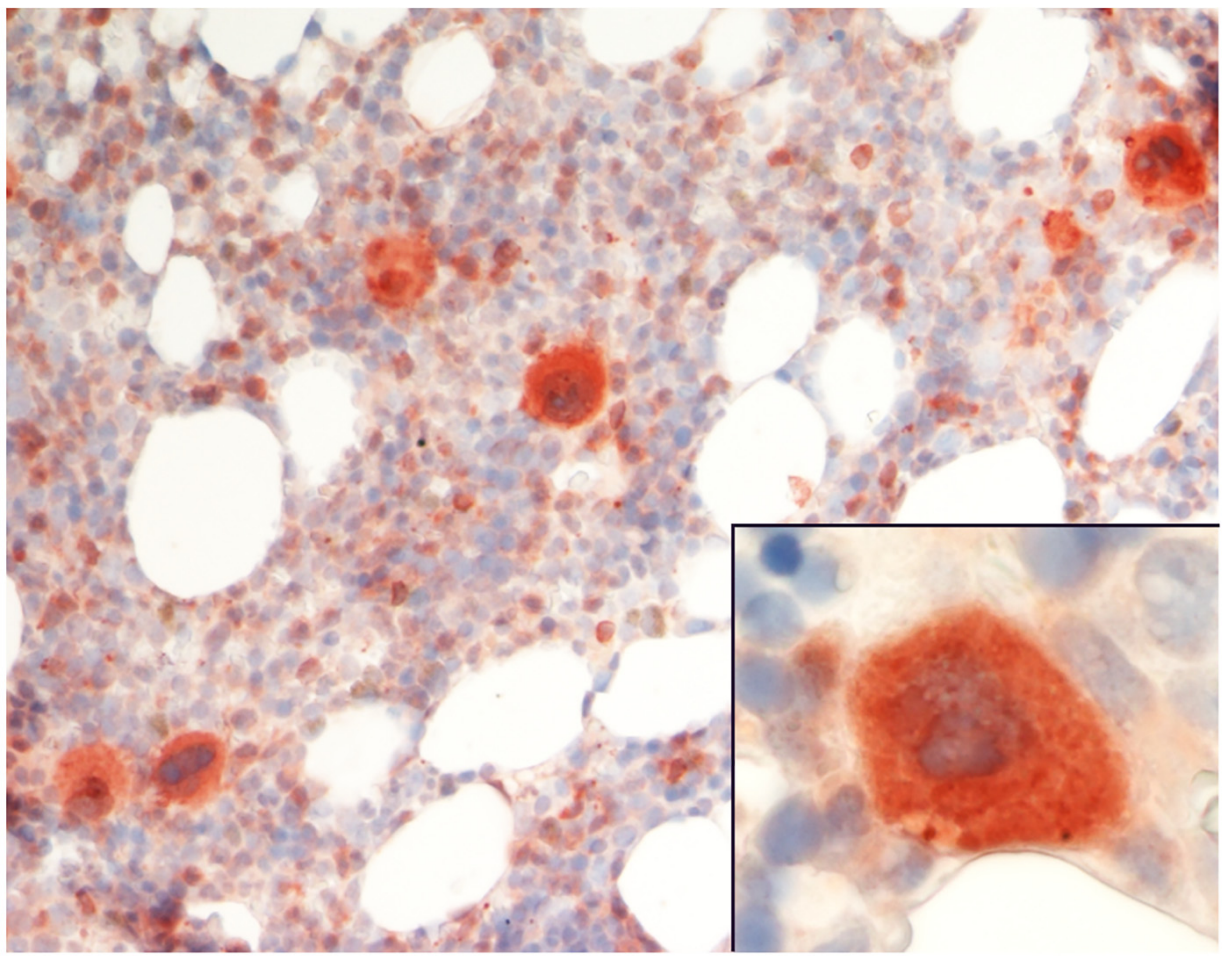
Expression of AZIN2 in megakaryocytes in normal bone marrow. Insert: A megakaryocyte at higher magnification showing a granular cytoplasmic staining.

In addition to our previously reported expression of AZIN2 in normal mast cells and in mastocytomas [21] we found AZIN2 in a slightly granular distribution in cytoplasm of megakaryocytes of normal bone marrow (Fig 2). Positive staining was also seen in aggregates of platelets and in mast cells / basophils.

### The Stomach

**Fig 3:**
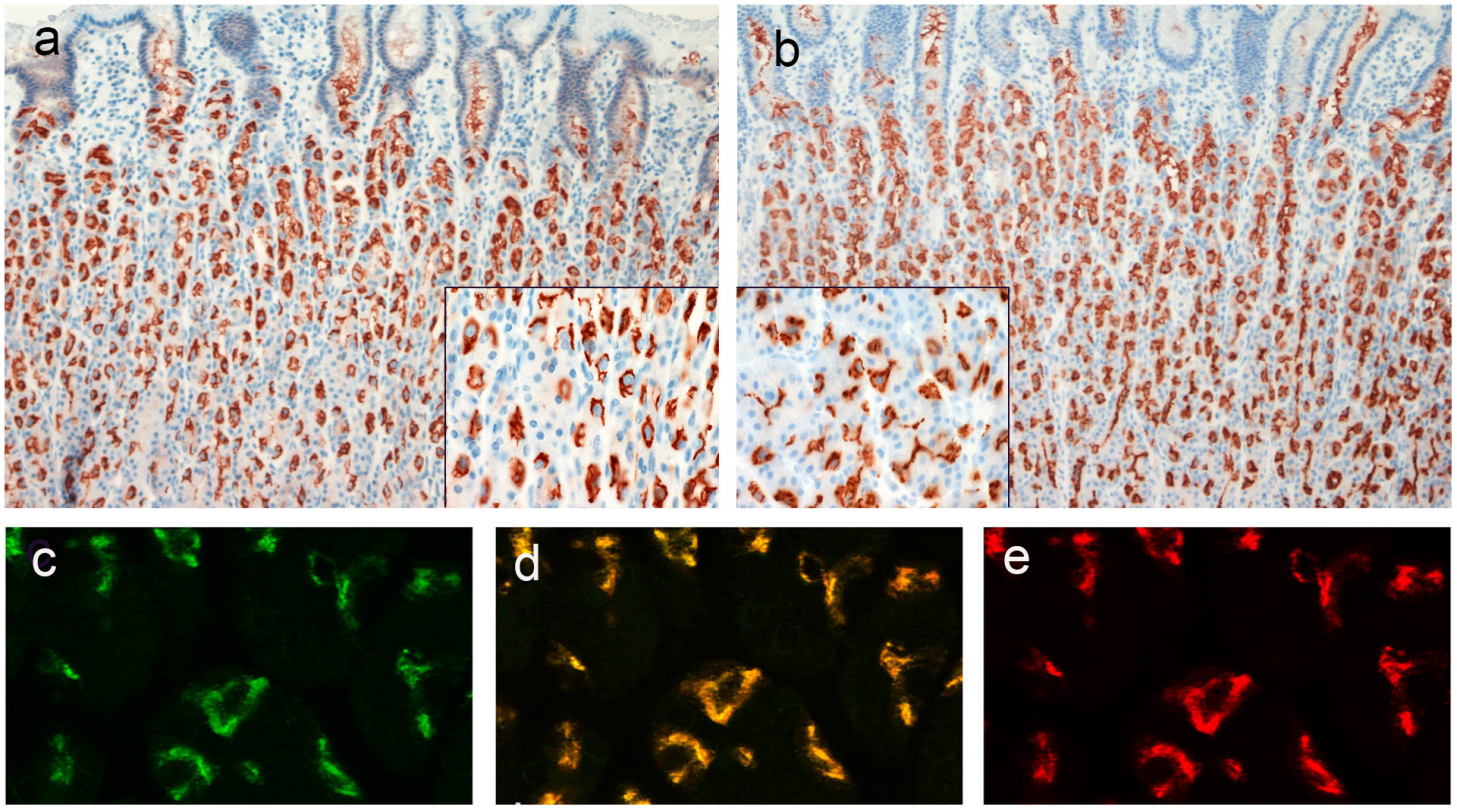
Staining of normal gastric mucosa (corpus). AZIN2 reactivity in a tubular pattern in parietal cells (insert) (a). A consecutive section stained with mAb to H,K-ATPase revealed a similar distribution (b). Confocal microscopy of double immunofluorescence staining of AZIN2 (e) and H,K-ATPase (c) show co-distribution in the merged picture (d).

A strong staining of AZIN2 in a tubular pattern was found in the parietal cells of normal gastric mucosa (Fig 3a). A staining of corresponding distribution was seen with a mAb to the H,K-ATPase beta subunit (Fig 3b). Confocal microscopy of double immunofluorescence staining showed co-distribution of AZIN2 and H,K-ATPase (Fig 3c–3e).

### Enteroendocrine Cells

**Fig 4:**
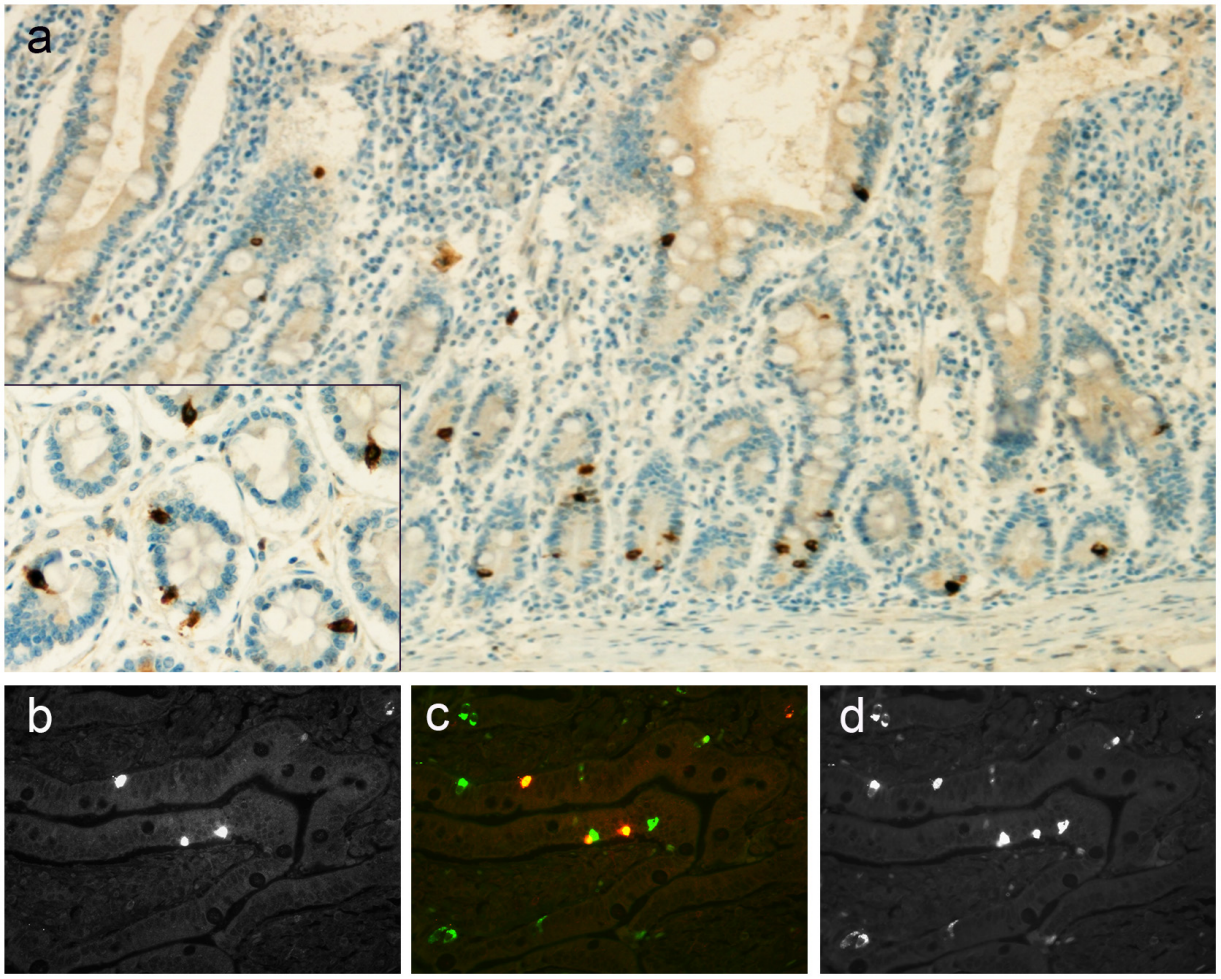
**Enteroendocrine cells** of the duodenal mucosa express AZIN2 (a) (insert at higher magnification). Double immunofluorescence staining with K3 (b) and mAb to ChrA (d) revealed a proportion of cells co-expressing AZIN2 and ChrA while some ChrA-positive cells remained negative for AZIN2 (merged picture c).

A proportion of enteroendocrine cells, particularly in the small intestine, showed strong staining for AZIN2 (Fig 4a). Double immunofluorescence with a mAb to the marker for enteroendocrine cells Chromogranin A (ChrA) revealed that about every third ChrA-positive cell co-stained for AZIN2 (Fig 4b–4d). Given our earlier finding that AZIN2 regulates secretion of serotonin but not of histamine from mast cells we performed a double immunofluorescence staining with a mAb to serotonin. Serotonin-positive enteroendocrine cells were seen but co-expression with AZIN2 was not detected (not shown). The endocrine product(s) elaborated by the AZIN2-positive cells in the intestinal mucosa remains to be identified.

### The Lung

**Fig 5:**
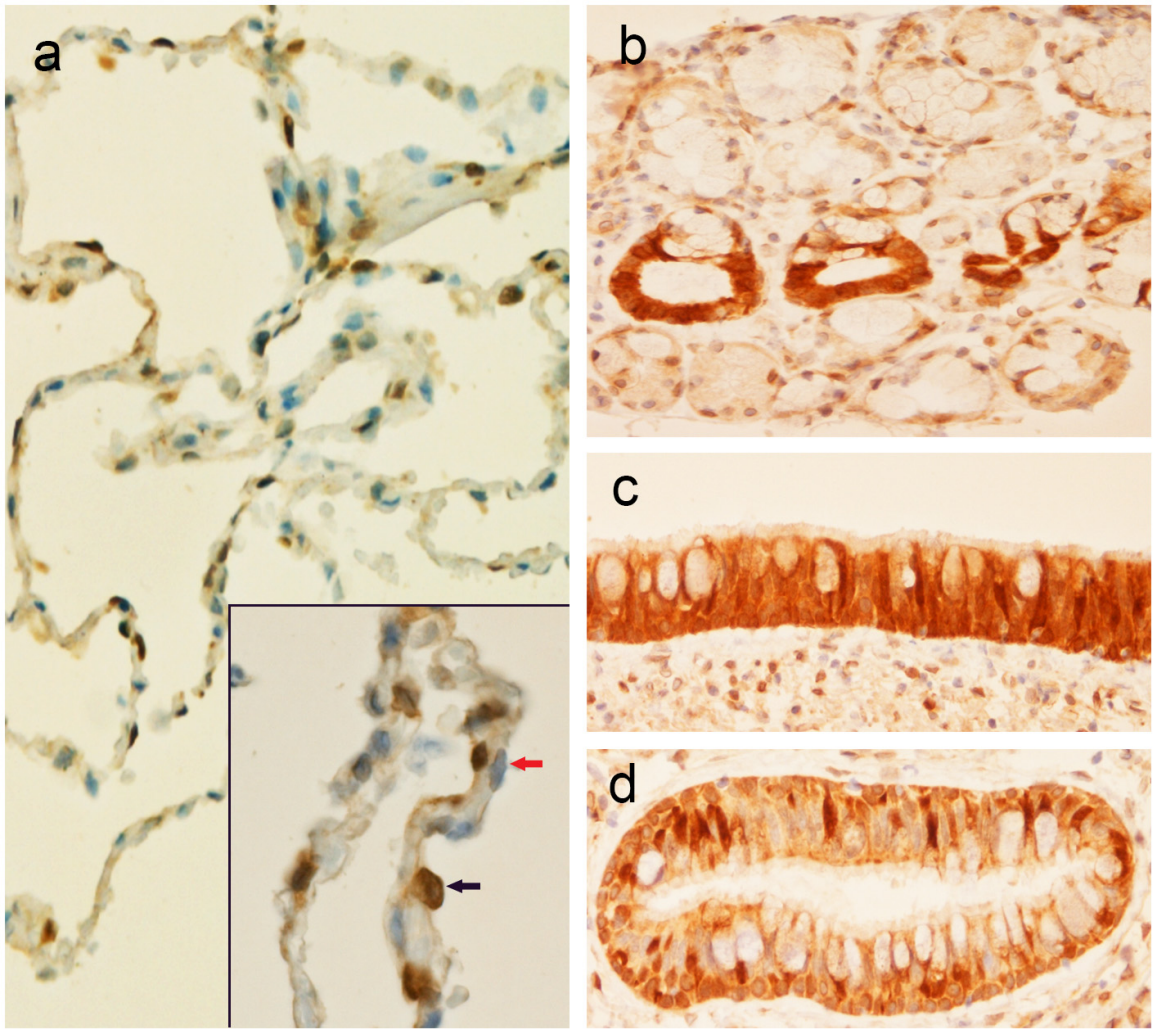
**Expression of AZIN2 in type 2 pneumocytes** (a, b black arrow) while type 1 pneumocytes are negative (a, b red arrow). Ductal epithelium of bronchial submucosal glands show strong staining in comparison to the acinar cells (c). Staining of AZIN2 in the mucosa of a large bronchus (d) and a smaller bronchus (e).

A robust expression of AZIN2 was found in type 2 alveolar pneumocytes that generate surfactant. No staining of AZIN2 was seen in type 1 alveolar pneumocytes (Fig 5a and 5b). The acinar epithelium of bronchial submucosal glands was negative while positive staining was seen in the ductal epithelium (Fig 5c).

The bronchial mucosa showed expression of AZIN2. A heterogenic staining pattern could be appreciated with particularly strong staining of the stalks of goblet cells while the content of the goblets remained negative (Fig 5d and 5e). Some distinctly staining cells close to the basal membrane were also seen. They may represent neuroendocrine (Kulchitshy) cells but identification of their phenotype needs additional studies.

### Exocrine Glands

**Fig 6:**
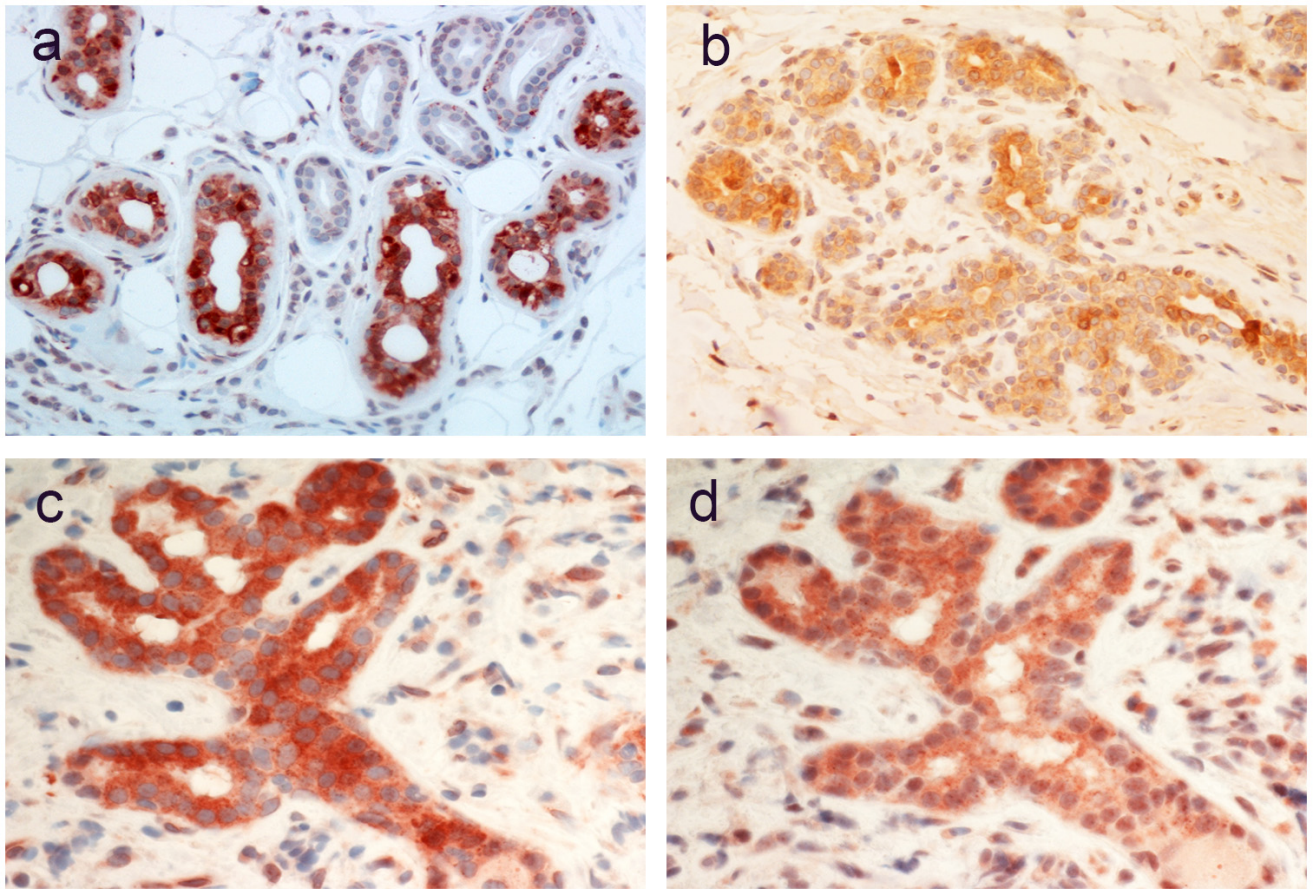
Expression of AZIN2 in exocrine glands. The sweat-producing acinar epithelium of a normal sweat gland shows strong AZIN2 expression while the ductal cells (upper right corner) are negative (a). Lobular and ductal epithelium in non-lactating breast shows a weak reactivity (b). Tear gland epithelium shows AZIN2 positivity (c) in a distribution similar to that seen for Rab27b (d).

The acinar, sweat-producing cells of normal sweat gland stained strongly for AZIN2. Viewing at high magnification revealed a granular cytoplasmic distribution of AZIN2. The epithelial cells of the ductal portion of the gland remained negative (Fig 6a).

Staining of breast tissue from non-lactating fertile women with morphological features of early luteal phase revealed weak staining of lobular epithelium with some more intensively stained individual cells in the ductal portions (Fig 6b). No appreciable expression of AZIN2 was seen in lobular epithelial cells of breast tissue representing an early follicular phase (not shown). The acinar cells of lacrimal glands showed positive staining (Fig 6c) with a distribution like that seen for the secretory G-protein Rab27b (Fig 6d).

### The Kidney

**Fig 7:**
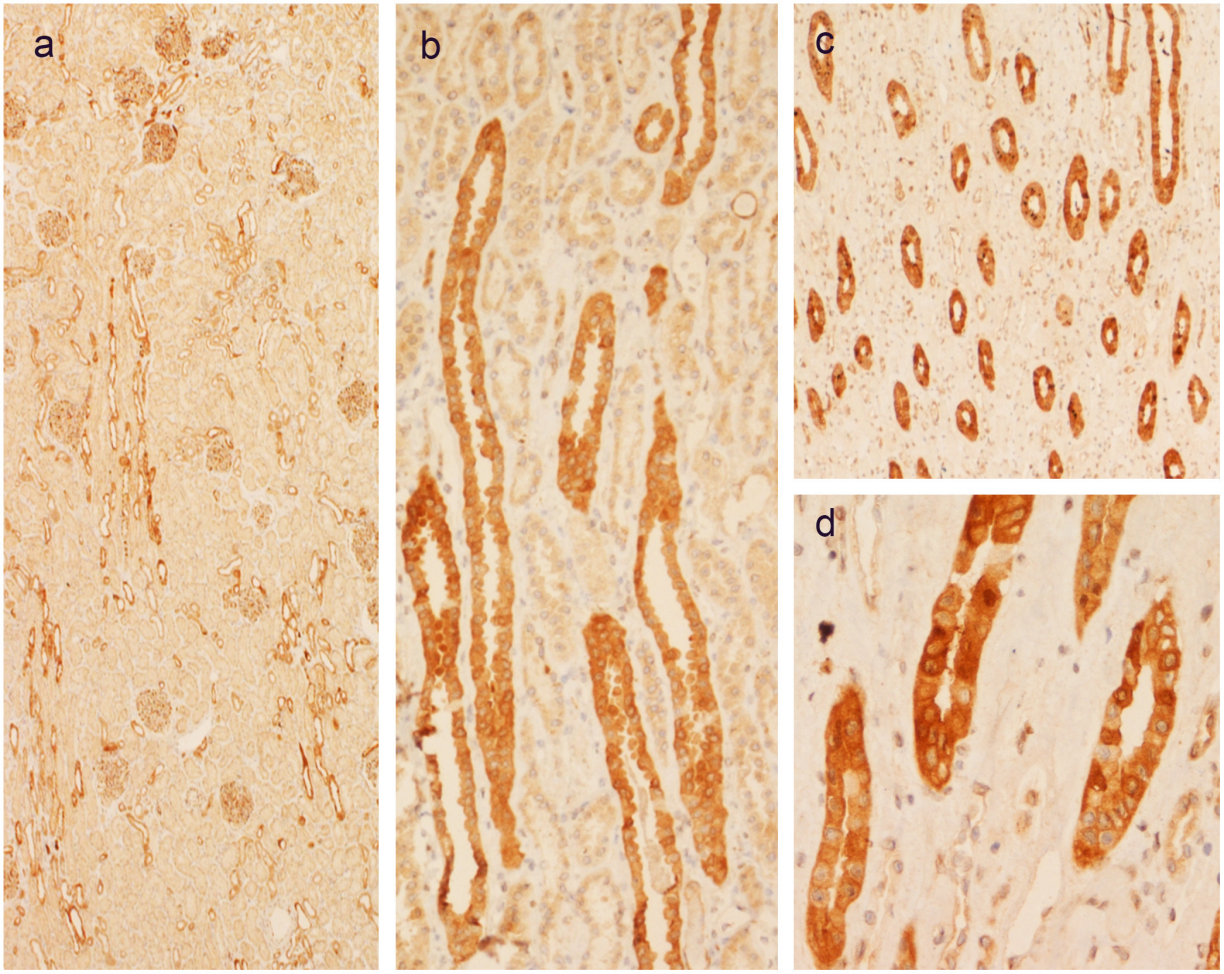
**Staining pattern for AZIN2 in kidney** corresponding to glomeruli and collecting ducts (a-c). Higher magnification shows variable staining intensity in adjacent epithelial cells of collecting ducts (d,).

A staining pattern corresponding to collecting ducts and glomeruli was seen in the kidney (Fig 7a). A heterogeneous staining intensity of individual cells was seen among the epithelial cells of colleting ducts (Fig 7b–7d). The phenotype of these cells remains to be characterized with additional markers.

**Fig 8:**
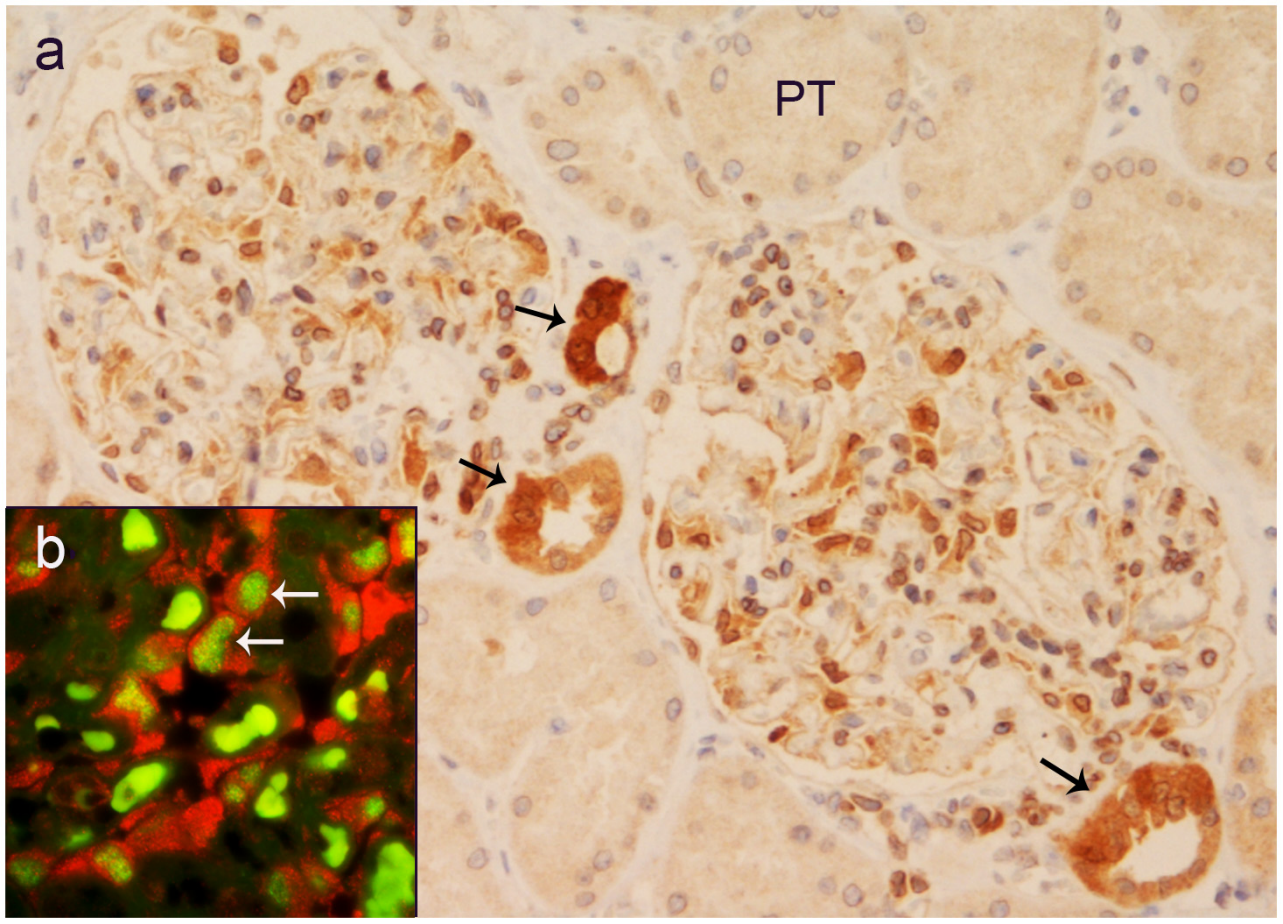
Selected glomerular cells show AZIN2 positivity. The high epithelium of *macula densa* (black arrows) contains abundantly of AZIN2 while proximal tubules (PT) are negative. Insert: Double immunofluorescence shows glomerular cells with cytoplasmic AZIN2 expression (red) and nuclear WT1 expression (green, white arrows). (Bright green background fluorescence in erythrocytes).

AZIN2-positive cells were present in the glomeruli. The epithelium of cortical proximal tubuli remained negative (Fig 8a). The distribution of the glomerular staining pattern suggested AZIN2 expression in podocytes. To identify the phenotype of the positively staining cells we performed double immunofluorescence with K3 antibodies and a mAb to WT1 as a nuclear marker of podocytes. A majority of the glomerular cells with cytoplasmic staining for AZIN2 also showed nuclear expression WT1 identifying them as podocytes (Fig 8 insert). The specialized juxtaglomerular epithelial cells of *macula densa* in cortical ascending tubuli showed a strong staining for AZIN2 (Fig 8a).

## Discussion

There are no previous reports on systematic mapping of the expression of AZIN2 protein in human tissues. The immunohistochemistry shown in this study is mainly based on an antibody called K3 that reacts with the splicing variants of AZIN2 containing exons 1 and 2. Given the high number of potential splicing variants [14] there may be tissues and cells expressing alternative splice variants not reactive with this antibody. In fact we found evidence for the expression of differently spliced forms of AZIN2 in the grey and white brain matter corresponding to the somas and dendrites of pyramidal neurons in brain neocortex [17]. This finding suggests that alternative splicing may guide compartmentalized targeting of AZIN2 expression.

The findings reported here show high physiological expression levels of AZIN2 in differentiated cells that display secretory activity or endowed with the molecular machinery needed for intracellular vesicle transport. This is particularly evident in acinar epithelium of sweat glands and in type 2 pneumocytes.

We found a strong expression of AZIN2 in gastric parietal cells co-localizing with the H,K-ATPase. The transport and secretion of gastric acid involves an intricate vesicular trafficking machinery. This includes several members of the Rab family of GTPases [23], SNARE proteins and secretory carrier membrane proteins. In addition to a regulatory influence on the vesicle transport it is tempting to speculate, that AZIN2 locally activates ODC that by decarboxylation of ornithine generates CO2 and hereby provides CO2 required for production of gastric acid [24].

The expression of AZIN2 in podocytes is in agreement with previous studies showing active release of vesicles from podocytes in the urine [25]. Impairment of vesicle trafficking by targeted deletion of the vacuolar sorting protein 34 in mouse podocytes has been shown to cause glomerulosclerosis [26] indicating the importance of an intact vesicle transport, where AZIN2 may be functionally involved.

The high epithelium in distal tubuli forming *macula densa* displayed a particularly robust staining. The cells sense changes in the NaCl concentration in the distal tubulus and elaborate prostaglandins that trigger release of renin from juxtaglomerular cells to regulate the glomerular filtration rate [27].

There was heterogenic staining intensity of AZIN2 in epithelial cell of collecting ducts. Whether the strongly staining cells are intercalated cells known to be rich in cytoplasmic vesicles [28] and the faintly staining cell principal cells remains to be established. Another possibility is that staining intensity mere reflects actual activity of the secretory or transport machinery of individual epithelial cells.

Viewing at high resolution revealed that the intracellular distribution of AZIN2 appears frequently in a granular or vesicular pattern. This is indicative of a spatial association between AZIN2 and secretory vesicles. The molecular mechanism(s) by which AZIN2 regulate vesicle transport remains to be elucidated. It appears however that local regulation of the catalytic activity of ODC and polyamine synthesis in distinct intracellular compartments is of importance. Small G-proteins, particularly of the large Rab family, are well-established regulators of vesicle transport [23].

We have previously reported evidence for compartmentalized activation of ODC during cell transformation and activation. We found that ODC activity regulates the intracellular traffic of RhoA during v-src-induced cell transformation. Locally generated putrescine activates RhoA via transglutaminase-catalyzed polyamination [29]. Since the ODC activity during cell transformation and proliferations is preferentially regulated via AZIN1 it is tempting to speculate that AZIN2 upregulates ODC activity in distinct subcellular compartments to yield polyamines that activate G-proteins of the Rab family via similar mechanisms. This hypothesis is currently under investigation.

The purpose of this report is to map the physiological expression of AZIN2 in selected normal cell and tissues. The main message indicates that polyamine metabolism regulated by AZIN2 plays a more compelling role in cellular secretory activity and /or vesicle transport than previously appreciated.
